# Impact of Blood Group on Contralateral Suppression of Otoacoustic Emissions, Speech Perception in Noise, and Binaural Integration

**DOI:** 10.1055/s-0046-1819712

**Published:** 2026-05-05

**Authors:** Pancham Ponnana S A, Jovita Priya Tauro, Nikhil Paremajalu S., Akhil Varghese, Kamalakannan Karupaiah, Prashanth Prabhu

**Affiliations:** 1 Department of Speech and Hearing, Father Muller College, Mangalore, Karnataka, India; 2 Department of Audiology, All India Institute of Speech and Hearing, University of Mysore's Research Centre, Mysore, Karnataka, India

**Keywords:** blood groups, contralateral suppression of OAE, efferent system, dichotic CV, binaural integration, speech in noise

## Abstract

**Introduction:**

Understanding the relationship between blood groups (A, B, AB, and O) and auditory functioning can provide valuable insights into individual differences in hearing ability and potential risks for hearing loss.

**Objective:**

To assess the impact of blood group on auditory functions. It examined contralateral suppression of transient evoked otoacoustic emissions (TEOAEs), speech perception in noise (SPIN), and binaural integration using the dichotic consonant-vowel (DCV) test.

**Methods:**

There were 60 female participants, from the blood groups A, B, AB, and O, aged 18 to 30 years with normal hearing were included. This study's preliminary assessments included pure-tone audiometry, immittance audiometry, and otoscopy to exclude hearing loss and middle ear dysfunction. Suppression of TEOAE was measured with and without contralateral broadband noise, while SPIN was tested at signal-to-noise ratios (SNRs) of −10, 0, and +10 dB. The DCV test assessed binaural integration with the presentation of prerecorded consonant-vowel stimuli simultaneously.

**Results:**

The Kruskal-Wallis H test showed no significant differences in TEOAE suppression amplitudes, SPIN scores across SNRs, or DCV scores across the four blood groups.

**Conclusion:**

These findings suggest that blood type has no discernible effect on central auditory functions, such as binaural integration, speech perception in noisy environments, or efferent auditory system functioning. This research, thus, highlights the need for further investigation with diverse methodologies and larger cohorts, to better understand the role of blood groups in auditory processing.

## Introduction

The ABO blood grouping system plays a crucial role in various physiological processes. It has been found to impact susceptibility and resistance to specific disorders, such as noise-induced hearing loss, sudden hearing loss, tinnitus, or presbycusis. 1 2 3 4 Understanding the relationship between blood groups and auditory performance can provide valuable insights into patients' differences and potential risks for hearing loss. This knowledge can help develop targeted interventions and preventive measures for individuals based on their blood groups, ultimately improving overall auditory performance.

By investigating the potential effects of different blood groups on auditory performance, we can gain a deeper understanding of the underlying mechanisms and pathways involved. Studies have indicated that individuals with the O blood group have a higher risk of developing disorders such as tinnitus, sudden hearing loss, or age-related hearing impairment. 1 2 3 4 5 6 7 The hematological function of the stria vascularis, which supplies blood to the cochlea, is also influenced by the ABO blood grouping system. 8

Understanding the specific mechanisms behind these potential associations is essential to develop targeted interventions. For instance, presuming that a certain blood group is associated with a higher risk of hearing loss, individuals with these blood types may benefit from early screening and proactive measures to protect their auditory performance. Furthermore, exploring this link can also shed light on the broader connections between the immune system, inflammation, and auditory system health. This interdisciplinary approach may lead to new strategies for preserving and improving auditory performance across diverse populations.

Overall, the need for studying the relationship between blood groups and auditory performance is driven by the potential to identify risk factors, develop targeted interventions, understand underlying biological processes, and improve the overall management of auditory disorders based on an individual's blood group. Additionally, this study may contribute to the broader field of personalized medicine, extending to other aspects of healthcare. This knowledge may contribute to advancements in the field of audiology, allowing for more precise diagnoses and tailored treatment options based on an individual's blood group.

Few studies have examined the impact of different blood types on auditory performance. Research employing otoacoustic emissions (OAEs) has demonstrated that, in contrast to other blood types, those with the O blood group experience a decrease in the amplitude of OAEs as well as a lower incidence of spontaneous OAEs. 2 9 Similarly, in infants with blood group O, spontaneous OAEs are less common, and the amplitude of Distortion Product OAEs is lower. 10 Studies on tympanometric results have revealed that, compared with other blood types, those with blood group O have higher resonance frequencies and relatively higher acoustic reflex thresholds. 11

A study examining the impact of blood groups on the Auditory Brainstem Response (ABR) showed that people with blood group O exhibited significantly lower amplitudes at lower repetition rates, and lower wave V amplitudes and latency at higher repetition rates. 8 Considering the previous studies, ABO blood grouping may have an impact on auditory performance. However, studies examining the impact of blood groupings on the operation of the efferent auditory system have not yet been conducted. Similarly, studies exploring the effect of blood groups on binaural integration are yet to be undertaken.

Therefore, the purpose of the current study was to compare the amplitudes of efferent suppression, scores of dichotic consonant-vowel (DCV) tests, and speech perception in noise (SPIN) scores across signal-to-noise ratios (SNRs) among various blood groups. This would, thus, help examine the effects of blood group on efferent auditory system functioning, binaural integration, and speech perception in noise.

## Methods

The present study was approved by the institutional ethics committee (Approval No: FMIEC/CCM/435/2020), and written informed consent was obtained from all the participants. The study involved a cohort of 60 female participants, all within the age range of 18 to 30 (mean age: 24 ± 3.74) years, with normal hearing abilities and varying blood groups. Prior to data collection, extensive preliminary assessments were conducted to ensure participant suitability. These included otoscopic examination, pure-tone audiometry, and immittance evaluation, aiming at excluding individuals with any signs of hearing loss or middle ear dysfunction.

Otoscopic examination, done using a standard otoscope, revealed a clear external auditory meatus with visibly intact tympanic membranes, with the cone of light visualized in both ears. Air conduction thresholds were determined utilizing a calibrated, two-channel clinical audiometer called the Inventis Piano (Inventis S.R.L.), equipped with Telephonics Dynamic Headphones 39 (TDH-39 - Telephonics Corporation) enclosed in MX-41/AR (Wilfan Electronics, Inc.) supra-aural ear cushions. Bone conduction thresholds were assessed employing the RadioEar B71 bone vibrator (RadioEar).

In terms of hearing criteria, normal hearing sensitivity (≤15 dBHL) across octave frequencies ranging from 250 to 8,000 Hz for air conduction and from 250 to 4,000 Hz for bone conduction were employed in pure-tone audiometry, along with the “modified Hughson-Westlake procedure” in both ears to exclude any peripheral hearing impairment. Following pure-tone audiometry, immittance evaluation was conducted utilizing a calibrated Inventis Clarinet (Inventis S.R.L.) middle ear analyzer to evaluate function. Tympanometry was performed using a probe tone of 226 Hz, and acoustic reflexes were assessed at 500, 1,000, 2,000, and 4,000 Hz using the quick mode (screening). All participants exhibited ‘A’ type tympanograms with acoustic reflexes at 500, 1,000, 2,000, and 4,000 Hz. Participants fulfilling the inclusion criteria after the preliminary investigations were selected for further investigations.

The core purpose of this research was to investigate contralateral suppression of Transient Evoked Otoacoustic Emissions (TEOAEs), SPIN using monosyllables, and DCV tests. For TEOAEs, contralateral suppression was measured using the Otodynamics Echoport ILO v6 (Otodynamics Ltd.) software in linear mode. Click trains were presented at 65 dB peak equivalent Sound Pressure Level (SPL) across test frequencies ranging from 1 to 5 kHz, employing 300 sweeps. Emissions were considered present if wave reproducibility exceeded 70% and the SNR was more significant than 6 dB at a minimum of three frequency bands.

To examine the Medial Olivocochlear (MOC) reflex, participants were exposed to contralateral continuous broadband white noise (range: 500–8,000 Hz) at 65 dB SPL, with masker on/off time set to 30 seconds, following the methodology outlined by Celikgun and Derinsu. 12 Amplitude reductions for each frequency were determined by subtracting emissions data recorded with contralateral white noise from emissions obtained without contralateral acoustic stimulation.

To assess speech perception, the SPIN test was conducted using a calibrated dual-channel Inventis Piano audiometer at −10 dB, 0 dB, and +10 dB SNR, with ipsilateral speech noise introduced. An HP laptop (Hewlett Packard Development Co., L.P.) computer with an Intel Core i3 (Intel Corp.) processor, externally connected to the audiometer, was used to present prerecorded monosyllables. These monosyllables were presented to each participant in two lists of 20 stimuli, each with a 1 second silence between two successive stimuli. 13

Participants' responses were manually recorded, and percentile scores were calculated to analyze speech perception in noise across different SNR conditions. Using the same dual-channel Inventis Piano audiometer that was calibrated for the SPIN test, the DCV test was conducted with a 0 milliseconds interaural delay between the ears. The audiometer was linked externally to the HP laptop, which was used to provide the stimuli. Participants received prerecorded consonant–vowel samples in both ears simultaneously and were instructed to record the syllables they perceived in each ear on a scoring sheet. The responses were manually recorded, and the outcomes were compared with existing normative data for further analysis.

### Statistical Analyses

The IBM SPSS Statistics for Windows (IBM Corp.), version 21.0, was used for the statistical analysis. To determine normality in the distribution of the data, the Shapiro-Wilk test was used. The outcomes showed that participants' data did not have a normal distribution. Hence, nonparametric statistical tests were used in the study.

## Results

The data's mean and standard deviation (SD) were determined using descriptive statistical analysis. The four blood types (A, B, AB, and O) were compared for contralateral suppression amplitudes across various frequencies, SPIN scores at three distinct SNRs, and DCV test scores using the Kruskal-Wallis H test for additional statistical comparison.

### Contralateral Suppression of TEOAE and Blood Group Findings

The contralateral suppression of TEOAE was analyzed using descriptive statistics. The amplitude reduction was similar across the different blood groups. These findings suggest no significant effect of blood group on OAE suppression.

To further validate this observation, Kruskal-Wallis H tests were performed for frequencies ranging from 1 to 5 kHz. The results indicated no statistically significant differences ( *p* > 0.05) across these frequency bands.

**Fig. 1:**
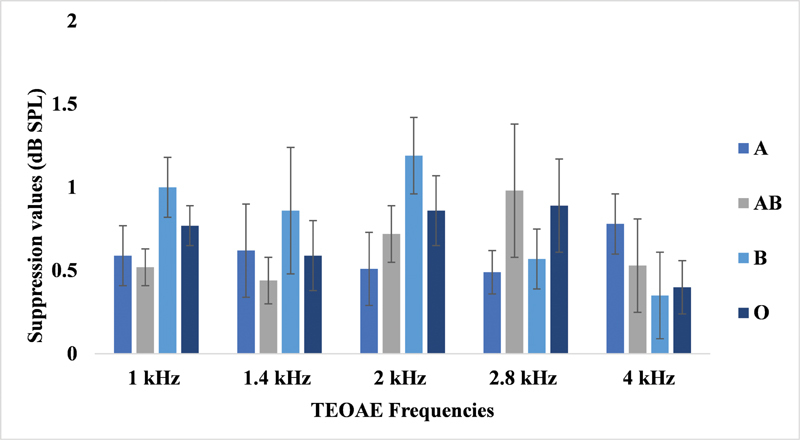
Contralateral suppression of transient evoked otoacoustic emissions across five frequencies for the four blood groups.

The mean and SD of the amplitude reduction (in dB) for contralateral suppression of TEOAE across blood groups at frequencies from 1 to 4 kHz are depicted in Fig. 1 .

### Blood Group Findings and SPIN

Similarly, the SPINtest results, evaluated at three different SNR levels (−10, 0, and +10 dB), were subjected to a descriptive statistical analysis. The scores were similar across all blood groups, indicating no significant differences in speech perception performance among individuals based on their blood group.

**Fig. 2:**
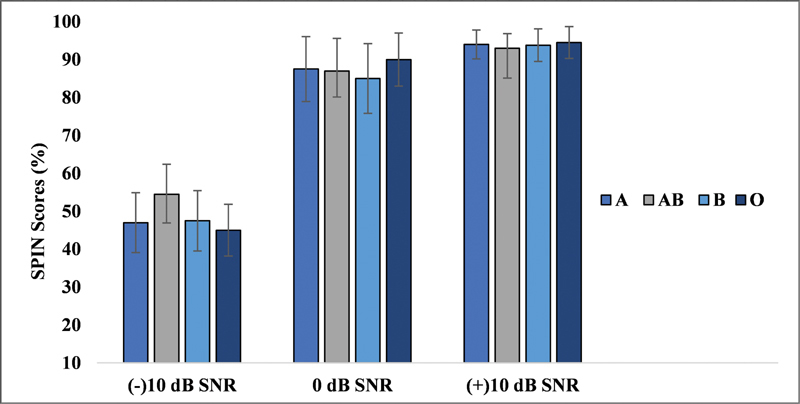
Speech perception in noise scores at three signal-to-noise ratios for all the four blood groups.

The mean and SD of the SPIN scores at each SNR for each blood group are provided in Fig. 2 .

Furthermore, a Kruskal-Wallis H test was used to examine whether the blood group had any significant effect on the SPIN scores at the different SNR levels. The results showed no statistically significant differences ( *p* > 0.05) at any of the SNR levels tested (−10, 0, and +10 dB). This indicates that the blood group does not have a measurable impact on SPIN at the given SNR levels.

### Blood Group Findings and DCV

The results of the DCV test were also subjected to descriptive statistical analysis. The single and double correct scores were found to be similar across the blood groups.

**Fig. 3:**
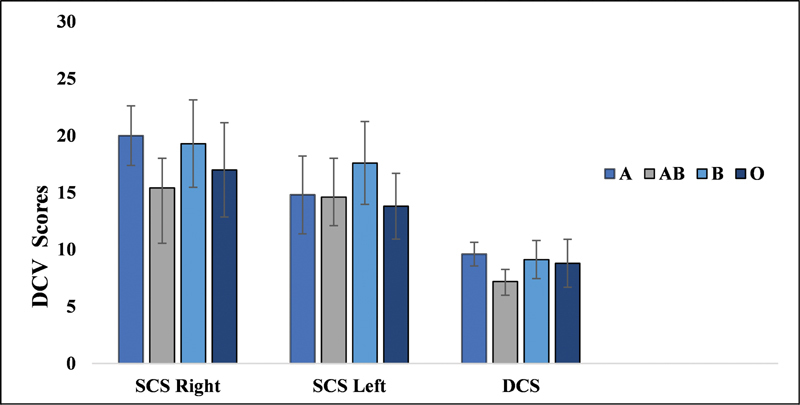
Dichotic consonant-vowel test scores for all the four blood groups.

The mean and SD of both single and double correct DCV test scores across blood groups are depicted in Fig 3 .

A subsequent Kruskal-Wallis H test was performed to assess whether any differences in the DCV scores existed between the blood groups. The results showed no statistically significant differences ( *p* > 0.05) in the groups' single and double correct scores. These findings suggest that the blood group did not influence performance on the dichotic listening task.

## Discussion

The current study aimed to examine whether changes in blood group affect the functioning of the efferent auditory system and crucial parameters assessing auditory performance, such as binaural integration and SPIN. Comprehending these correlations is key, since previous studies have predominantly concentrated on genetic, environmental, and physiological elements impacting auditory performance, with the effect of blood type going relatively unexplored.

To address this gap, the Kruskal-Wallis H test was employed to compare contralateral suppression amplitudes across frequencies, SPIN scores at three SNRs, and DCV scores among the four major blood groups (A, B, AB, and O). However, contrary to our hypothesis, the analysis revealed no statistically significant differences ( *p* > 0.05) among the blood groups for any of the auditory tests conducted. These findings align with those of Mo et al. 14 , Kumaar et al. 15 , and Kara et al., 16 who examined the impact of blood groups on OAE, such as distortion product otoacoustic emissions (DPOAEs) and transient evoked otoacoustic emissions (TEOAEs). Their findings also indicate no significant differences in amplitudes of otoacoustic emissions among different blood groups, supporting the hypothesis that blood group may not be a critical factor affecting auditory performance, particularly in relation to efferent auditory pathways.

However, these results are in contrast with previous findings, suggesting that blood groups might influence different domains of auditory performance. For instance, individuals with blood group O were previously shown to have decreased amplitudes and a reduced incidence of spontaneous OAEs. 2 9 Similar outcomes were reported by Li et al., who found that infants in this blood group had smaller DPOAE amplitudes and fewer spontaneous OAEs overall. 10 Additionally, blood group O individuals demonstrated higher resonance frequencies, elevated acoustic reflex thresholds, 11 and reduced ABR amplitudes, along with prolonged wave V latencies, suggesting potential disruptions in neural auditory processing.

One possible explanation for the discrepancy between our findings and those of earlier studies is the nature of the auditory assessments employed. While the current study assessed higher-level auditory performance parameters, such as contralateral suppression of TEOAEs, SPIN, and binaural integration, previous research predominantly focused on peripheral auditory mechanisms, including OAEs and tympanometry. 17 Thus, it is plausible that blood group may exert a more pronounced effect on peripheral auditory function than on central auditory processing, which involves more complex neural mechanisms.

Additionally, differences in demographic characteristics and sample sizes may have contributed to the divergent findings. Notably, earlier studies often involved neonates or narrowly defined populations, which may be more susceptible to subtle effects of blood group. In contrast, the current study included a broader and possibly more heterogeneous population, potentially diluting minor group-based effects. Taken together, the results of this study suggest that blood group does not need to be considered when evaluating contralateral suppression of TEOAEs, SPIN, or binaural integration. This finding has clinical implications, as it may streamline audiological assessments by allowing clinicians to focus on more relevant influencing factors.

Although the current study found no significant evidence to suggest that blood type, including group O, significantly affects certain auditory processes, prior findings indicate that this potential influence should not be entirely dismissed. Therefore, future studies are encouraged to explore other biological or genetic elements that may influence auditory processing. Moreover, targeted investigations involving specific populations or auditory subsystems may help clarify whether blood group exerts a more nuanced effect under certain conditions.

## Conclusion

The present study's findings did not reveal any statistically significant differences among blood groups in the amplitudes of contralateral TEOAE suppression, SPIN scores, or DCV test scores. This suggests that the blood group does not affect the ability to perceive speech in noisy environments, binaural integration, or efferent system performance at the frequencies often utilized in audiometry. This study is limited by its sample size and the inclusion of only female participants. Future research should include a more diverse population and age groups, to evaluate plausible fluctuations in efferent system performance across the lifespan and explore longitudinal effects. Subsequent investigations have to contemplate employing ultra-high frequencies for assessing contralateral suppression. Enhancing generalizability also requires repeating the study with a larger sample size.
